# Cognitive behavioral therapy for insomnia in Parkinson’s disease: a case series

**DOI:** 10.1038/s41531-017-0027-z

**Published:** 2017-07-28

**Authors:** Meghan Humbert, James Findley, Maria Hernandez-Con, Lana M. Chahine

**Affiliations:** 1 Lake Erie College of Osteopathic Medicine, Greensburg, PA USA; 2 Department of Sleep Medicine, University of Pennsylvania Perelman School of Medicine, Philadelphia, PA USA; 3 Department of Neurology, University of Pennsylvania Perelman School of Medicine, Philadelphia, PA USA

## Abstract

Chronic insomnia is common in patients with Parkinson’s disease. There are limited data to guide its treatment in this patient population, especially in regards to non-pharmacologic interventions, some of which are highly effective in the non-Parkinson’s disease population. The aim of this study is to describe a series of Parkinson’s disease patients who underwent cognitive behavioral therapy for insomnia (CBTi). Parkinson’s disease patients who had undergone a baseline and at least one follow-up CBTi session were identified. Electronic medical records and pre-treatment and post-treatment patient sleep diaries were reviewed. Sleep measures of interest included wake time after sleep onset, sleep efficiency, sleep onset latency, and total sleep time. Pre-treatment and post-treatment values were compared within subjects using paired *t*-test. Five patients were included. Patients attended an average of eight sessions of CBTi (range 5–12). Significant increases in sleep efficiency (*p* = 0.02) and decreases in number of awakenings per night (*p* = 0.02) were found. Our data provide preliminary evidence that cognitive behavioral therapy is an effective treatment for insomnia in Parkinson’s disease, and is well tolerated and well received by patients. Given the limited data supporting use of medications to treat chronic insomnia in Parkinson’s disease, combined with their risks, randomized trials to demonstrate the efficacy of CBTi in Parkinson’s disease are warranted.

## Introduction

Sleep disturbances are among the most common non-motor symptoms (NMS) in Parkinson’s disease (PD).^1^ Insomnia, defined as trouble falling or staying asleep with daytime sequelae, is the most commonly reported sleep disturbance,^2^ occurring in 55–80% of patients.^1, 3^ Insomnia significantly negatively impacts quality of life in PD.^4–9^ Thus, effective and safe therapies for it are essential. Evidence supporting the use of hypnotics for treatment of chronic insomnia in PD is limited, and there are significant concerns for side effects, particularly increased fall risk.^10^ Therefore, non-pharmacologic therapies are desirable. Cognitive behavioral therapy for insomnia (CBTi) has proven efficacy in the treatment of insomnia in non-PD populations,^11–13^ but has not been well studied in individuals with PD. We herein report a series of patients with PD treated with CBTi.

## Results

A search of the University of Pennsylvania Health System (UPHS) electronic medical record (EMR) system on January 16, 2016 yielded eight patients who had been referred for CBTi and had a PD diagnosis. Five patients completed at least one follow-up visit and were included in this report. Patient demographics, PD history, other clinical history, and sleep history are shown in Table 1. Self-report measures pre- CBTi and post-CBTi are also shown in Table 1. A summary of each case follows.

**Table 1:** (a) Demographics/clinical characteristics and sleep history (b) Patient-reported measures of sleep following the baseline assessment and prior to initiation of CBTi (pre-CBTi) and the last week of CBTi (post-CBTi)

Variable	Patient 1	Patient 2	Patient 3	Patient 4	Patient 5
(A) Demographics and clinical characteristics					
Age (years)	69	68	39	30	55
Sex	Male	Female	Male	Female	Female
Body mass index	27.6	25	34	27.9	36.6
Parkinson’s disease duration at CBTi (years)	5	2	2	1	1
PD symptoms	Severe resting/kinetic tremor, with mild rigidity and bradykinesia. Good response to dopaminergic medications	Mild parkinsonism, manifesting with tremor and bradykinesia. Good response to dopaminergic medications	Mild tremor and bradykinesia. Good response to dopaminergic medications	Mild tremor and bradykinesia. Painful dystonic episodes in right foot. Good response to dopaminergic medications	Moderate tremor and bradykinesia, severe rigidity. Good response to dopaminergic medications
Sleep co-morbidities	None	Obstructive sleep apnea Delayed sleep–wake phase disorder	REM sleep behavior disorder controlled on clonapzeam	None	Obstructive sleep apnea
Medical Co-morbidities	Diabetes History of prostate cancer Depression, controlled on medication	Diabetes Hypercholesterolinemia Degenerative disc disease	Degenerative disc disease Thyroid cancer History of infectious encephalitis (10 years prior to PD symptom onset) Right lower extremity radicular pain controlled on duloxetine	Asthma	Depression Hypertension Chronic low back pain Nephrolithiasis Leg claudication of unclear etiology (extensive workup)
Relevant medications at time of CBTi	Amantadine 100 mg three times a day Carbidopa/levodopa 25/100 mg, 1,5 tablets four times a day Escitalopram 10 mg daily	Carbidopa/levodopa 25/100 mg, 1 tablet three times a day	Clonazepam 1 mg at bedtime Duloxetine 30 mg daily Ropinirole XL 16 mg po daily	Carbidopa/levodopa 25/100 mg, 1 tablet every 4 h 5 times a day Rasagiline 1 mg daily Ropinirole XL 4 mg daily	Carbidopa/levodopa 25/100 mg, 2 tablets three times a day Bupropion XL 150 mg daily
Use of hypnotics at baseline	Zolpidem Trazodone	None	Trazodone	None	None
Self-reported bedtime (prior to CBTi)	11:30 PM–12:00 AM	12:30–1:30 AM	10:00 PM–1:00 AM	10:00–11:00 PM	9:00 PM on weeknights, 10:00 PM on weekends.
Self-reported terminal awakening time (time of day the patient wakes up and starts their da) (prior to CBTi)	8:30–9:00 AM	7:30–8:00 AM	6:00 AM on weekdays and Saturdays 8:00 AM on Sundays	7:00–7:30 AM	6:00 AM
Perpetuating factors for insomnia: maladaptive behaviors and environmental Characteristics	Daytime napping Excessive mentation in bed at bedtime and during awakenings Feelings of frustration when unable to sleep	TV-watching in bed Cats in the bedroom. Excessive mentation at bedtime and during awakenings Increased attention to sleep and sleep related stimuli	Nocturnal computer use TV on overnight in bedroom. Excessive mentation at bedtime and during awakenings	Nocturnal computer use TV-watching in bed Excessive mentation at bedtime and during awakenings	Reading in bed Smartphone use in bed Excessive mentation at bedtime/during awakenings, feeling of anxiety near bedtime/during awakenings
Self-reported psychosocial contributors to insomnia	Financial	Health-related concerns	Family/Children/Parents	Work	Health-related concerns
Caffeine, alcohol, and nicotine use	Caffeine. No alcohol intake. Remote ex-smoking.	No caffeine. Remote ex-smoker. Rare wine intake.	No caffeine or alcohol intake	“Rare” tea intake. No history of smoking. No alcohol intake.	No caffeine or alcohol intake. Ex-smoker
Daytime sleep	Unintentional dozing and intentional napping during the day of variable durations	Unintentional dozing during the day, of variable duration	Intentional napping up to 2 h during the day on weekends.	Intentional daytime naps during the day of 60 min duration, on 3 days/week.	Unintentional dozing of 1–2 h during the day
(B) Patient-reported measures of sleep pre-CBTi and post-CBTi					
Number of in-person CBTi visits	6	8	12	7	5
Duration of CBTi therapy (days)	72	120	127	57	51
Mean sleep onset latency pre (minutes)	107	62	39	26	69
Mean sleep onset latency post (minutes)	14	27	10	10	33
Mean WASO pre (minutes)	87	7	27	121	19
Mean WASO post (minutes)	26	7	24	37	36
Maximum number of awakenings after sleep onset in a week	2	3	4	2	3
Maximum number of awakenings after sleep onset in a week**	1	3	2	1	2
Mean TST pre (minutes)	301	378	236	330	233
Mean TST post (minutes)*	429	423	408	422	210
Mean Sleep efficiency pre	62	84	74	67	64
Mean Sleep efficiency post**	93	96	92	80	67
Number of nights rated poor (0, 1, 2 (quality)) pre	3	1	2	2	0
Number of nights rated poor (0, 1, 2 (quality)) post	0	0	2	4	2

Values shown are mean values from the patient diaries for the 7 days pre-CBTi and the last available 7-day diary data that the patient recorded post-CBTi

*CBTi* cognitive behavioral therapy for insomnia, *TST* total sleep time, *WASO* wake time after sleep onset

* 0.05 < *p* < 0.10 (paired *t*-test comparing post CBTi to pre CBTi value)

**p < 0.05 (paired *t*-test comparing post CBTi to pre CBTi value)

*Patient #1*: At baseline CBTi visit, this was a 69-year-old male with a 5-year history of PD. He had a chronic history of difficulty initiating sleep, which had worsened dramatically during the year prior to CBTi, forcing him to retire from his truck-driving job. He denied motor complaints affecting his sleep, including no history of restless leg syndrome (RLS) symptoms. He felt unrefreshed on awakening.

**Fig. 1:**
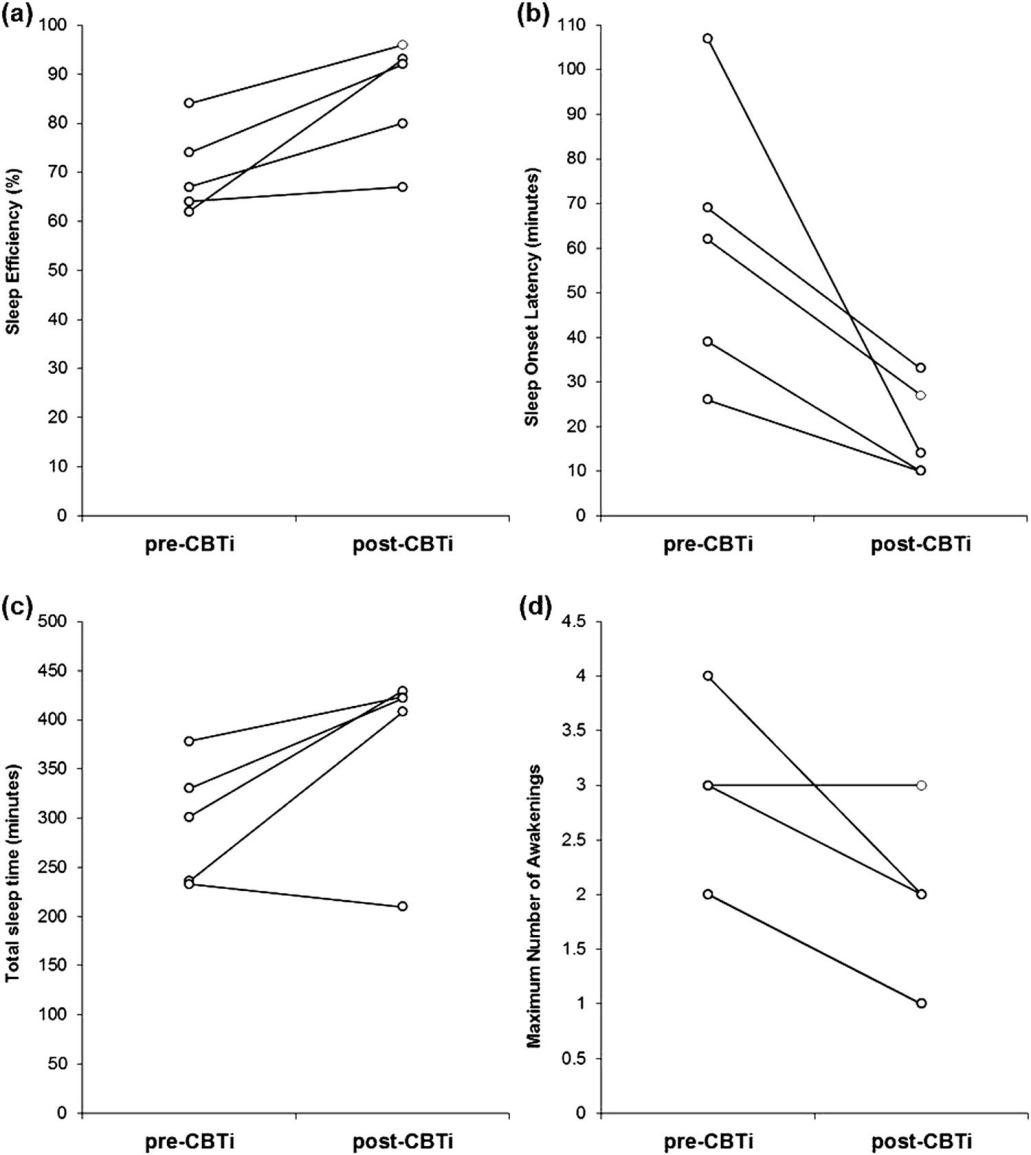
Within-subject changes post-CBTi compared to pre-CBTI in the following self-reported sleep measures: **a** sleep-efficiency **b** sleep onset latency **c** total sleep time **d** maximum number of nocturnal awakenings

Course of therapy/outcome: he attended six CBTi sessions, with dramatic improvements in wake after sleep onset (WASO) time (70% reduction) and sleep efficiency (SE) (50% improvement) (Fig. 1; Table 1).

*Patient #2*: At baseline CBTi visit, this was a 66-year-old female with a 2-year history of PD who reported a decades-long difficulty falling asleep. The patient described herself as a “night-owl”. She did not perceive her motor symptoms as contributing to nocturnal sleep problems. She denied RLS symptoms. She reported snoring. During a one-night in-lab polysomnogram, she had an apnea–hypopnea index (AHI) = 7.8, REM–AHI = 15.4, no periodic limb movements (PLMs), and an arousal index (AI; number of EEG arousals per hour) of 11.3. The patient declined continuous positive airway pressure therapy for her mild obstructive sleep apnea (OSA). Since her main complaint was difficulty in initiating sleep, she was referred for CBTi despite her co-morbid untreated OSA. She reported waking up feeling unrefreshed. She was diagnosed with both sleep maintenance insomnia as well as delayed sleep–wake phase disorder.

Course of therapy/outcome: she attended eight CBTi sessions. Following completion of therapy, she reported being able to fall asleep at a more desirable bedtime for her (11:45 PM), with a sleep onset latency (SOL) reduction of 85% (Fig. 1; Table 1), and woke up feeling more refreshed.

*Patient #3*: This was a 39-year-old male with a 2-year history of PD at baseline CBTi visit. He reported chronic snoring and a 2-year history of difficulty initiating and maintaining sleep which began soon after his PD diagnosis. One-night in-lab polysomnography showed AHI = 3.8, no PLMs, and AI = 10.4. He reported feeling unrefreshed on awakening. Of note, he admitted to compulsive behaviors likely related to exposure to dopamine agonists, including over-eating and prolonged nocturnal computer.

Course of therapy/outcome: he attended 12 CBTi sessions. By the end of therapy he had a 75% reduction in SOL (Table 1). His WASO time was not significantly changed.

*Patient #4*: This was a 30-year-old female with a 1-year history of PD at baseline CBTi visit. She had a history of painful but levodopa-responsive episodes of right foot dystonia that began at the age of 19. At age 29, she developed parkinsonism, responsive to dopaminergic therapy, as well as motor fluctuations and dyskinesias. Dopamine transporter (DAT) SPECT scan showed reduced DAT binding, consistent with nigrostriatal denervation, which in the context of her clinical picture, was felt to be consistent with PD despite her young age. She reported occasional painful nocturnal leg cramps, but otherwise did not feel her motor manifestations contributed to her insomnia. Her main sleep complaint was difficulty maintaining sleep that had started 5 years prior to CBTi referral. One-night in-lab polysomnography showed AHI = 0.3, no PLMs, and AI = 24.1. She reported an average of 2 prolonged nocturnal awakenings/night.

Course of therapy/outcome: She attended seven CBTi sessions. At the end of CBTi she had achieved a 75% reduction in WASO time and 35% increase in total sleep time (TST) (Fig. 1; Table 1).

*Patient #5*: At baseline CBTi visit this was a 55-year-old female with a 1-year history of PD. She endorsed a 10-year difficulty of falling asleep as well as multiple nocturnal awakenings. She reported occasional nocturnal leg cramps, but otherwise denied nocturnal pain or motor symptoms affecting her sleep, and denied RLS symptoms. She reported snoring and frequent episodes of dream enactment. One-night in-lab polysomnography showed AHI = 4.0, REM AHI = 8.1, no PLMs, and AI = 11.3. She reported multiple awakenings per night.

Course of therapy/outcome: She attended five CBTi counseling sessions. Despite good compliance with CBTi recommendations, TST and WASO did not improve (Fig. 1; Table 1). While quantitative measures of her sleep did not improve, she did report a subjective benefit from CBTi.

## Discussion

This case series provides preliminary evidence that CBTi can be an effective treatment option for individuals with insomnia and PD. CBTi significantly improved sleep efficiency and subjective sleep quality in 4/5 patients.

Sleep disturbances are among the most commonly cited NMS in PD. Commonly occurring sleep disorders in PD include REM sleep behavior disorder, OSA, PLM disorder, and RLS.^3^ In patients with more advanced disease, tremor, rigidity, pain, and impaired bed mobility contribute to difficulty in sleeping, and PD medications can adversely affect sleep as well.^3, 14^ Neuropsychiatric symptoms may also account for sleep problems in some PD patients. Thus, PD sleep problems are likely multifactorial. However, in many PD patients with complaints of difficulty initiating and maintaining sleep, identifiable contributing factors and/or and or sleep disorders (besides insomnia) are either not found or do not fully explain the severity of the sleep complaints. The degree to which intrinsic abnormalities in central nervous system structure and function account for sleep problems in such cases is poorly understood, but degeneration of brainstem/thalamocortical regions involved in the sleep-wake cycle likely contribute to sleep abnormalities in PD, as do abnormalities in the suprachiasmatic nucleus and other hypothalamic structures.^15^ The contribution of poor sleep hygiene, environmental sleep disorder, and psychophysiological insomnia to sleep disturbances in PD have not been well studied. In the patients we presented, nocturnal motor symptoms, RLS, and neuropsychiatric symptoms did not seem to contribute significantly to the patients’ insomnia. Several of the patients reported maladaptive sleep-related behaviors and poor sleep hygiene, which are addressed by CBTi.

There are limited evidence-based therapies for insomnia in PD.^3^ One small study^16^ over a 6-week period enrolled six patients in one of three study arms (i) non-pharmacologic intervention, combining CBTi with light therapy (ii) doxepin 10 mg and (iii) placebo. At 6 weeks, insomnia severity score and subjective sleep quality significantly improved in the CBTi group compared to placebo. While patients in our case series were not administered validated scales of sleep quality, information obtained from their EMR, as well as the sleep diaries, also showed evidence of subjective improvement in sleep quality. There are otherwise limited data on the utility of CBTi in PD. CBT has been administered to individuals with PD for treatment of various other disorders including anxiety,^17, 18^ depression,^17, 18^ and impulse control disorders.^19^ Data from those studies as well as our experience suggest that this patient population is amenable to CBT, compliant with visits and adherent to CBT recommendations. It is notable that in our case series, there was a wide range of age, disease duration, various co-morbidities, and different sleep symptoms and disorders, suggesting that CBTi may be useful in the relatively heterogeneous PD patient population. Having said that, there are several potential barriers to administration of CBTi in PD. These include the above-mentioned contributors to sleep problems in PD as well as cognitive dysfunction which may limit understanding of and compliance with CBTi. Which patient subgroups are most suitable for CBTi, and what the barriers are for its application in this patient population, requires further study.

Our data are limited by the retrospective nature of this study, the small sample size, and lack of a non-disease control group for comparison. In addition, validated objective and subjective sleep measures as outcomes for the intervention were not available. However, participants filled out comprehensive sleep diaries, which allowed for analysis of quantitative measures of self-reported sleep pre- and post-CBTi. The findings in this case series provides compelling data supporting the investigation, in randomized controlled trials, of CBTi in PD.

## Methods

### Subject identification and data collection

This was a single-center, retrospective case series. Inclusion criteria: (i) age >18, (ii) clinical diagnosis of parkinsonism presumed to be due to a neurodegenerative disorder and (iii) one baseline and at least one follow-up visit with a cognitive behavioral therapist that included documentation of sleep-related data (see below). After Institutional Review Board approval, the UPHS EMR system was queried for individuals with an International Classification of Disorders-9 code for PD who had been seen at both UPHS’ PD and Movement Disorders Center and the Sleep Medicine Center. The EMR of those meeting inclusion criteria were reviewed.

Data were collected from patients’ EMR and sleep diaries, and included patient demographics, medical (including neurologic) and social history, sleep disorder history, CBTi treatment intervention, and outcome. Methods were performed in accordance with relevant regulations and guidelines.

### Intervention

At the UPHS Sleep Center, CBTi is administered by individuals (typically with PhD in Psychology or nurses) certified in Behavioral Sleep Medicine.^20^ The CBTi program is tailored to the patient’s specific condition/needs but includes some combination of sleep hygiene education, stimulus control, relaxation training, and sleep restriction.^21, 22^ Sleep hygiene education involves the teaching of healthy practices to improve sleep. The goal of stimulus control is to reduce and ultimately eliminate any association of the bed with wakefulness/waking activities. This technique involves the patient remaining in bed for no longer than an estimated 20 min at a time. The patient is instructed to get out of bed (for 30 min) if they estimate they have been awake for more than 20 min. Time duration estimates, with avoidance of clock watching, is emphasized. Relaxation training involves teaching patients such techniques as progressive muscle relaxation, guided imagery, and/or abdominal breathing. Sleep restriction involves assigned bed times that initially limit time spent in bed, with the goal of enhancing sleep drive and decreasing time spent in bed awake (once sleep efficiency improves, sleep opportunity is upwardly titrated in 15–30 min increments).

Patients included in this study met with a provider qualified to administer CBTi for a baseline visit in which the above techniques were discussed and a treatment strategy was established. Patients followed a weekly schedule in which they filled out a sleep diary and documented their best estimate of time in bed, time to sleep, total (number and duration of) awakenings, and early morning awakenings. Patients also described their overall sleep quality on a 1–5 scale, 1 = very poor and 5 = very good. Patients met with the provider on a weekly basis to discuss their past week’s sleep habits and to set the treatment plan for the following week. Over the course of therapy, weekly adjustments to the administered CBTi techniques occurred as needed.

Variables used to asses efficacy of CBTi (collected or derived from sleep diary data) included: SOL, number of awakenings after sleep onset, WASO (time spent awake after initial period of sleep), TST, SE (time spent asleep/time spent in bed), subjective sleep quality, and subjective daytime impairment.

### Statistical analysis

Demographics and clinical characteristics were summarized and tabulated. Paired-samples *t*-test was applied to asses for differences in sleep measures post-CBTi vs. pre-CBTi using Stata version-13.0 (College Station, TX: StataCorp).

### Data availability

All relevant data are available from the authors at request.
